# Primary functional brain connections associated with melancholic major depressive disorder and modulation by antidepressants

**DOI:** 10.1038/s41598-020-60527-z

**Published:** 2020-02-26

**Authors:** Naho Ichikawa, Giuseppe Lisi, Noriaki Yahata, Go Okada, Masahiro Takamura, Ryu-ichiro Hashimoto, Takashi Yamada, Makiko Yamada, Tetsuya Suhara, Sho Moriguchi, Masaru Mimura, Yujiro Yoshihara, Hidehiko Takahashi, Kiyoto Kasai, Nobumasa Kato, Shigeto Yamawaki, Ben Seymour, Mitsuo Kawato, Jun Morimoto, Yasumasa Okamoto

**Affiliations:** 1 Department of Psychiatry and Neurosciences, Graduate School of Biomedical Sciences, Hiroshima University, Hiroshima, Japan; 2 ATR Brain Information Communication Research Laboratory Group, Kyoto, Japan; 3 Institute for Quantum Life Science, National Institutes for Quantum and Radiological Science and Technology, Chiba, Japan; 4 Medical Institute of Developmental Disabilities Research, Showa University, Tokyo, Japan; 5 Department of Functional Brain Imaging Research, National Institutes for Quantum and Radiological Science and Technology, Chiba, Japan; 6 Department of Neuropsychiatry, Keio University School of Medicine, Tokyo, Japan; 7 Department of Psychiatry, Kyoto University Graduate School of Medicine, Kyoto, Japan; 8 Graduate School of Medical and Dental Sciences, Tokyo Medical and Dental University, Tokyo, Japan; 9 Department of Youth Mental Health, Graduate School of Medicine, The University of Tokyo, Tokyo, Japan; 10 Computational and Biological Learning Lab, Cambridge University, Cambridge, UK

**Keywords:** Biomarkers, Depression, Biomarkers, Neuroscience

## Abstract

The limited efficacy of available antidepressant therapies may be due to how they affect the underlying brain network. The purpose of this study was to develop a melancholic MDD biomarker to identify critically important functional connections (FCs), and explore their association to treatments. Resting state fMRI data of 130 individuals (65 melancholic major depressive disorder (MDD) patients, 65 healthy controls) were included to build a melancholic MDD classifier, and 10 FCs were selected by our sparse machine learning algorithm. This biomarker generalized to a drug-free independent cohort of melancholic MDD, and did not generalize to other MDD subtypes or other psychiatric disorders. Moreover, we found that antidepressants had a heterogeneous effect on the identified FCs of 25 melancholic MDDs. In particular, it did impact the FC between left dorsolateral prefrontal cortex (DLPFC)/inferior frontal gyrus (IFG) and posterior cingulate cortex (PCC)/precuneus, ranked as the second ‘most important’ FC based on the biomarker weights, whilst other eight FCs were normalized. Given that left DLPFC has been proposed as an explicit target of depression treatments, this suggest that the limited efficacy of antidepressants might be compensated by combining therapies with targeted treatment as an optimized approach in the future.

## Introduction

Major depressive disorder remains a major global health challenge, with substantial socio-economic cost. The mainstay of pharmacological treatment is selective serotonin reuptake inhibitors (SSRIs) and serotonin-norepinephrine reuptake inhibitor (SNRIs). Despite their widespread and increasing use^1–4^, many patients respond little, if at all^5,6^. Understanding why this is the case is complicated, because of the poorly understood relationship between the regionally distributed actions of drugs and the complex underlying neurobiology of depressive symptoms.

Recently, brain imaging has provided important insights into the underlying neural mechanisms of depression. In particular, resting-state connectivity studies in humans have identified a number of potentially important abnormal functional connections that may play a role in disorder symptoms^7–14^. However, according to the meta-analysis paper of MRI-based neuroimaging biomarkers in depressive disorders^15^, around 30% of reviewed papers were using resting-state fMRI (rsfMRI) as modality, and only one-third of them were using functional connections (FCs) among region of interests (ROIs) as features. As most of those biomarker studies applied the algorithm of support vector machine (SVM) to achieve high accuracy with many features, it has not yet been clear about which are the most critical FCs in depression with whole-brain analysis.

In order to determine target FCs and investigate their modulation by antidepressants, it would be important to focus on a specific subtype of depression, because of heterogeneity of depression. Melancholic major depressive disorder (MDD) is a subtype of MDD that is traditionally considered to be the most drug-responsive, and so provides an ideal target to probe the effect of drugs on abnormal connectivity^16–20^. Reliably identifying drug effects on connectivity requires a robust biomarker that directly maps connectivity patterns to depressive symptoms.

As hypotheses on modulation by antidepressants, one possibility is that abnormal connectivity in MDD is uniformly but only partially resolved following treatment. On the other hand, it may be that whilst some functional connections fully resolve, others do not, or are even worsened by treatment. This latter possibility is particularly intriguing since it would suggest that the relative effect on different functional connections determines treatment response, as well as identifying specific targets for future combined treatment approaches. Therefore, the purpose of this study is to develop a melancholic MDD biomarker based on functional connections and explore the sensitivity to treatment.

In theory, this could identify functional connections which would be potential targets of depression treatment. This is because, first, the resting state functional connectivity is temporal correlations between two brain regions of interests (ROIs), and it is flexibly changed based on the type of cognitive tasks and easily can be targeted in training or intervention treatment studies in a short period of time. Second, there have been more and more interests in studying the network brain activity in fMRI data. The main functional networks are default mode network (DMN), executive control network (ECN), and salience network (SN). Specifically, DMN is observed as the network of regions functionally connected with each other during rest (i.e., with correlations of spontaneous temporal fluctuations of BOLD signals), and it is known to be correlated with depression symptom severity in recurrent MDDs^21^. So, some critical FCs in DMN could be both a good diagnostic measure and a good treatment target. Third, it has been reported that the change of within-DMN functional connectivity extends to other regions in the default mode modules, and also associated with FCs in the fronto-parietal module^22^. This suggests the possibility that we may be able to target and focus on only a critically abnormal FC to normalize, in order to affect the whole DMN to reduce depressive symptoms. At the same time, however, it is important to point out that functional connectivity studies are purely correlative even for the predictive diagnosis classification, and it should be difficult to disentangle causal versus consequential changes which track behavior and symptoms. To the best of our knowledge, there is no answer yet to the question if abnormality of functional connection is a cause of depression or an epiphenomenon caused by depression. However, based on the following reasons, we think that it would be appropriate to target the functional connection which is diagnostically most reliable. Although there is a limitation that we cannot really know if the correlational relationship could be causal, more and more researchers are focusing on the brain networks and abnormality of neural circuit dynamics, as a key for successful analysis to integrate different levels of knowledge into a comprehensive system^23–25^. Before such prospects, much more work is needed in fact, and we hope our work can contribute to the movement.

Our aims in this study were therefore two-fold: first, to extract critically important functional connections when building a classifier of melancholic MDD; and second, to use it to test the uniformity versus heterogeneity connectivity hypotheses of the effect of antidepressants on melancholic MDD patients.

## Methods

### Participants and clinical measures

**Figure 1:**
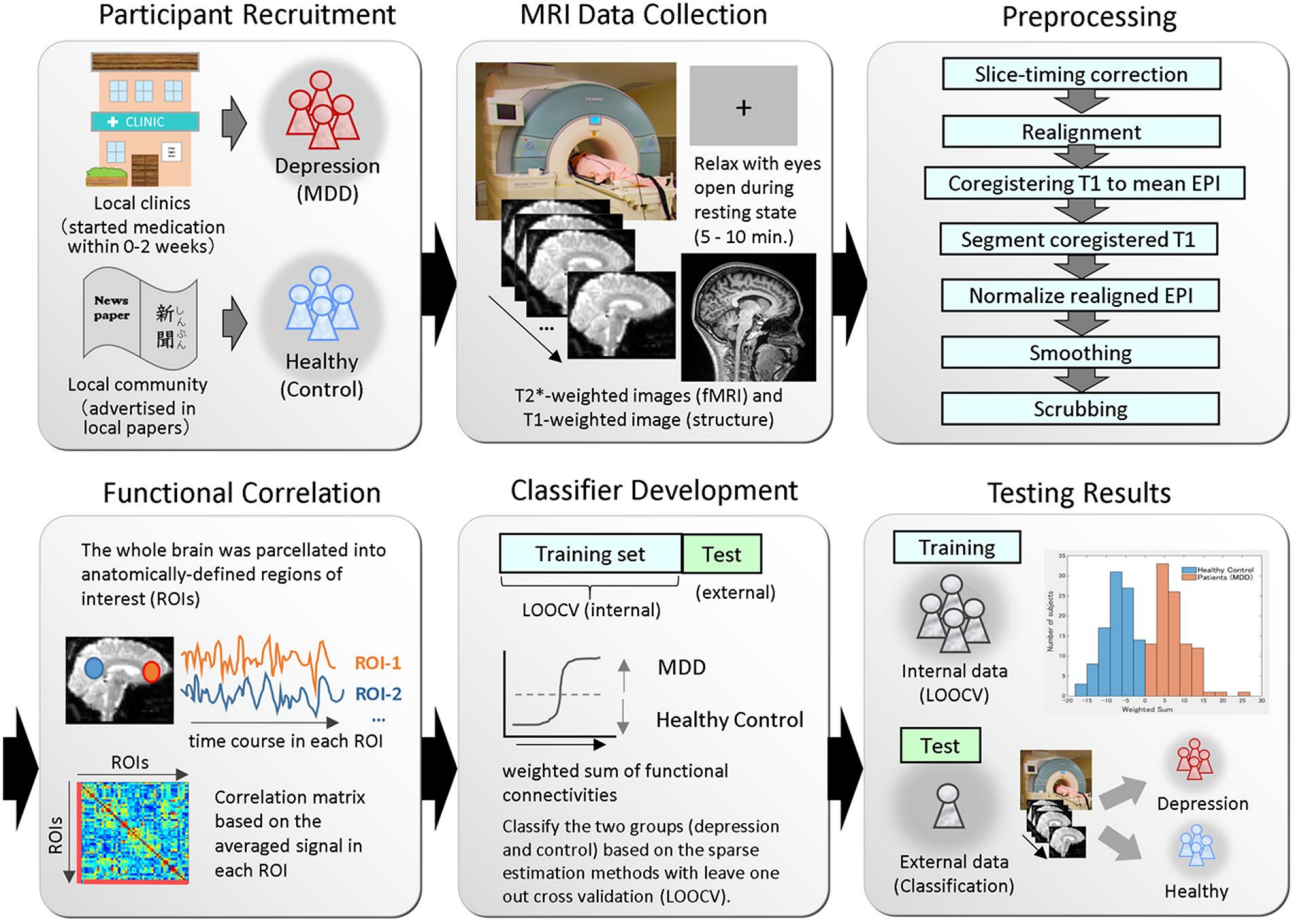
The integrated pipeline for depression biomarker development. Patients were recruited from local clinics. Resting-state fMRI and structural T1 data were collected using the identical scanner for each subject. Neuroimaging data analysis including preprocessing, functional correlation, and classifier development were performed by integrated scripts.

The overview of depression biomarker development is shown in Fig. 1. 177 patients were recruited at the Hiroshima University Hospital and local clinics (in Hiroshima, Japan) and screened using the M.I.N.I.^26,27^ for a MDD diagnosis with the DSM-IV criteria. Out of them, 118 patients participated in the MRI experiments. Exclusion criteria included current or past manic episodes; psychotic episodes; alcohol dependence or/and abuse; substance dependence or/and abuse; and antisocial personality disorder based on M.I.N.I., change of diagnosis (from unipolar to bipolar depression), MRI scan after more than 2 weeks of medication, fMRI data with excessive head motions based on scrubbing results (see the following section of Neuroimaging data preprocessing and interregional correlations). In addition, as the exclusion criteria at the moment of recruitment at local clinics included: already enough amount of dose and time of one type of antidepressant was administered, more than 2 types of antidepressants were administered for the current episode, had electroconvulsive therapy (ECT), physical disorders which may have any negative effects with SSRI treatments, current pregnancy or breast-feeding, high risk of suicidality judged by the doctor, those who needed to be hospitalized, and those who were not able to understand Japanese expressions. 92 patients with more than mild depressive symptoms (i.e., BDI >= 17) were included in the dataset of training the MDD classifier in the end. Patients had an initial MRI scan before or after starting medication within 0–2 weeks. 171 healthy controls (HCs) were recruited from the local community, interviewed with the M.I.N.I., and none showed any history of psychiatric disorders. Ten subjects out of 165 HCs who participated in the MRI experiment were excluded based on M.I.N.I. and on the quality check of MRI. The data of age and sex matched 92 HCs with no or low depressive symptoms (i.e., BDI <= 10) were used for training classifiers for all MDDs. For development of melancholic MDD biomarker, the data of 65 melancholic MDD patients out of 92 MDDs, and age and sex matched 65 HCs were included (Table 1 and Supplementary Table S1a). The number of patients and healthy controls were set to be equal, in order to develop a classifier unbiased toward either group. Prior to the administration of any experimental procedure, written informed consent was obtained from all participants. For the training dataset of the all MDD classifier, we used all the melancholic and non-melancholic MDD data collected from four different sites to evaluate the entire heterogeneous depression cohort. For the melancholic MDD classifier, the training dataset was limited to have the subtype of melancholia (based on M.I.N.I.) with moderate depression symptoms+ for patients based on the Beck Depression Inventory^28^ (BDI-II score 17 or higher). For scores of the Japanese version of national adult reading test (JART)^29^, which was used to estimate the intelligence quotient (IQ), there were eleven missing data in HCs of the training dataset. Additional details on clinical populations can be found in the Supplementary Table S1a–c. The experiments were carried out in accordance with relevant guidelines and regulations, all our experimental protocols were approved by the Ethics Committee of Hiroshima University, and informed consent was obtained from all subjects.

**Table 1:** Demographic and clinical information of the subject of melancholic MDD classifier.

	Melancholic MDD	Healthy Control	P-value
No. of participants	65	65	NA
Sex (Male/Female)	38/27	32/33	*p* = *0.29*
Age (Mean (SD))	43.7 (12.8)	43.6 (10.2)	*p* = *0.92*
Self-rating depression scale(BDI-II, Mean (SD))	31.2 (8.2)	3.6 (3.1)	*p* = 0.000***
Observer-rated depression scale (HAMD-17, Mean (SD))	19.9 (5.2)	N/A	NA
IQ (JART, Mean (SD))	108.3 (10.6)	111.9 (8.6)	*p* = 0.047*
Handedness (left/right)	0/65	0/65	NA
Melancholia (No.%)	100	0	NA

The differences between melancholic MDD and healthy controls were evaluated by two-tailed t-test for age, BDI-II, and JART respectively, whereas it was evaluated by chi-squared test for sex. ***p < *0.001*, *p < *0.05*.

### Measures of depression and depressive symptoms

The Beck Depression Inventory (BDI) is one of the most frequently used instruments for measuring depression and depressive symptoms as a self-rating scale. Additionally, the Hamilton Rating Scale for Depression (HAMD)^30^ was used to assess the pharmacological treatment effects as an observer rating scale.

### Experimental protocol and data acquisition

The following common instructions and settings were used in all the sites. In the scan room with dimmed lights, participants were required to keep looking at a cross mark in the center of the monitor screen, think of nothing in particular, and not to sleep. Details of scan parameters for MRI data acquisition and procedure in each site were shown in Supplementary Table S2.

### Neuroimaging data preprocessing and interregional correlations

T1-weighted structural image and resting state functional images were preprocessed using SPM8 (Wellcome Trust Centre for Neuroimaging, University College London, UK) on Matlab R2014a (Mathworks inc., Massachusetts, USA). The functional images were preprocessed with slice-timing correction and realignment to the mean image. Then, using the normalization parameters obtained through the segmentation of the structural image aligned with the mean functional image, the fMRI data was normalized and resampled in 2 × 2 × 2 mm^3^ voxels. Finally, the functional images were smoothed with an isotropic 6 mm full-width half-maximum Gaussian kernel. After these preprocessing steps, a scrubbing procedure^31^ was performed to exclude any volume (i.e., functional image) with excessive head motions, based on the frame-to-frame relative changes in time series data. In order to keep data quality high enough for the subsequent analyses, we only included the subject data with more than 50% of the volumes survived in the time series (see Supplementary Table S3 for a summary of head motion).

For each individual, the time course of fMRI data was extracted for each of 137 regions of interests (ROIs), anatomically defined in the Brainvisa Sulci Atlas (BSA; http://brainvisa)^32,33^ covering the entire cerebral cortex. We did not incorporate the cerebellum in the construction of a classifier, because for many participants in site 1, the cerebellum was truncated in their structural and functional images. After applying a band-pass filter (0.008–0.1 Hz), the following nine parameters were linearly regressed out: the six head motion parameters from realignment; the temporal fluctuation of the white matter; that of the cerebrospinal fluid; and that of the entire brain. A pair-wise Pearson correlations between 137 ROIs were calculated to obtain a matrix of 9,316 FCs for each participant. Details of classification algorithm for FC selections were described in the following section.

### Classification algorithm for FC selections

By combining the following two machine learning algorithms, we developed a melancholic MDD classifier with identification of characteristic FCs. As the first algorithm, L1-regularized sparse canonical correlation analysis (L1-SCCA)^34^ was applied to reduce the number of features to exclude the effects of nuisance variables (NVs) that may cause catastrophic over-fitting. L1-SCCA excluded the effects of nuisance variables (NVs) including sex, age, and scanner type (i.e., four different scanners with two different manufacturers; see the subsection of Supplementary Information entitled “Feature selection with reduction of nuisance variable effects” for details). As the second algorithm, sparse logistic regression (SLR)^35^ was applied to identify small number of features with high contribution to classification. The SLR algorithm decides the final number of features based on the principle of Automatic Relevance Determination (ARD)^36^, completely automatically without any meta-parameter. The method uses a sequential process of nested-feature selection and leave-one-out cross validation (LOOCV) in order to avoid information leakage and over-optimistic results^5^. Our machine learning algorithm was a combination of nested cross validation and LOOCV. In order to have more than twenty subjects per fold, we used 6-fold CV for the melancholic MDD classifier development. At the end of LOOCV, the output of the logistic regression classifier was used to compute the classification accuracy. The stability and robustness of the selection of the FCs in the LOOCV by evaluating their cumulative absolute weights ${c}^{k}=\mathop{\sum }\limits_{i=1}^{N}|{w}_{i}^{k}|,$ where *N* is the number of LOOCV folds (i.e. the number of subjects), and *w*^*k*^_*i*_ is the weight associated with the *k*-th FC during the *i*-th LOOCV fold. The greater magnitude of *c*^*k*^ indicates a more significant contribution by the *k*-th FC to the classification into melancholic MDD and HC, throughout the LOOCV. The more detailed description of this algorithm with some figures are found in the methods section of the autism spectrum disorder paper^37^. The original code developed for the autism spectrum disorder classification is available as well (for access, please contact the server administrator of ATR Brain Information Communication Research Laboratory: http://asd-classifier@atr.jp).

### Generalization to independent cohorts for validation

An independent external validation cohort was formed at the National Institute of Radiological Sciences in Chiba, Japan. Based on M.I.N.I., the participants (n = 51, Supplementary Table S1b) were evaluated on lifetime history of psychiatric disorders. None of the MDD patients (n = 11) had comorbid psychiatric disorders, and none of the healthy controls (n = 40) had any somatic, neurological, or psychiatric disorders and no history of current or previous drug abuse. All the participants were antidepressant and antipsychotic drug-free for more than 1 month on the day of MRI scan. All participants provided written informed consent before the study. The study protocol was approved by the Radiation Drug Safety Committee and by the institutional review board of the National Institute of Radiological Sciences, in line with the ethical standards established in the 1964 Declaration of Helsinki and its later amendments. Details of generalization to other psychiatric disorders and statistical methods on examining the changes of each FC with antidepressant treatments are shown in Supplementary Information.

### Pharmacological treatment effects

Twenty-five patients with melancholic depression in the training dataset had an additional MRI scan after 6 weeks of treatment with antidepressants (SSRI; see Supplementary Table S1c). Because this is an observational study and not a clinical trial, it was not possible to have a placebo or no treatment control group. Although it was a critical issue, based on our hypothesis, we applied the classifier to this post-treatment dataset to examine if all the identified functional connections would be uniformly resolved following treatment, or whilst some functional connections fully resolve, others do not, or are even worsened by the pharmacological treatments.

## Results

### Classification of melancholic MDD and generalization to an independent cohort

Classification was first performed by feature-selection from all 9,316 connections using L1-SCCA and SLR, and then the weighted linear summation (WLS, linear discriminant function) of the identified functional connections was evaluated. First, we tried this algorithm to all MDD (n = 184). However, a leave-one-out cross validation (LOOCV) revealed a classification accuracy of only 60% (AUC 0.62, sensitivity 59%, specificity 62%). Next, we focused on a more drug-responsive subtype, melancholic MDD. Then, the LOOCV result on the melancholic MDD (n = 130, Table 1) revealed a classification accuracy of 84% (AUC 0.91, sensitivity 80%, specificity 88%; *p* = *0.002* with a permutation test). We then tested the classifier on an independent cohort from a different site (n = 51), which revealed an accuracy of 69% (AUC 0.69, sensitivity 64%, specificity 70%; *p* = *0.040* with a permutation test, Supplementary Fig. S1a–d). Together, even with the limitation of small sample size, these results provided initial evidence that the classifier was moderately generalizable.

### Functional connections underlying classification

**Figure 2:**
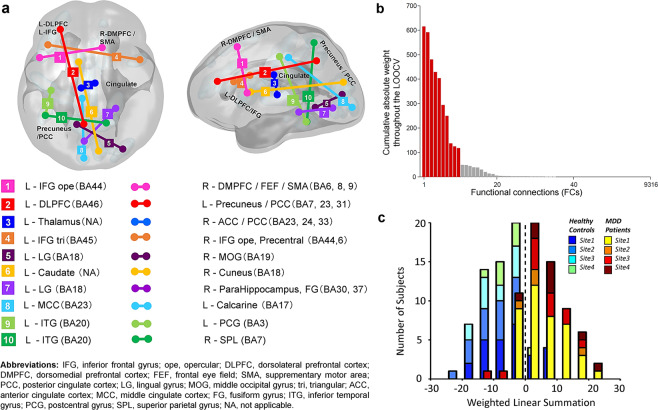
The identified 10 functional connections for the melancholic MDD biomarker. (**a**) Brain regions included in the identified functional connections (FCs) of the melancholic MDD classifier. Cumulative absolute weight of 32 FCs in total, which were selected at least once throughout the LOOCV. The identified 10 FCs are shown in red, and the rest of 22 FCs are in Gray. (**c**) Distribution of each subject’s weighted linear sum (WLS) was colored by site, in order to check for any potential scanner or site related effect (melancholic MDD: n = 65, HC: n = 65).

We then examined the neural basis of melancholic MDD classifier. The sparse classification algorithm identified 10 functional connections (FCs) as listed in Table 2. Ten FCs were sorted based on its contribution level. The ROIs included in the identified FCs are shown on a glass brain in Fig. 2a. In the distribution of the cumulative absolute weight (Fig. 2b, see Methods), we found that the identified 10 FCs were indeed the top 10 most important FCs among the 32 FCs that were selected at least once during the LOOCV, indicating the reliability of the FC selection in the present procedure. It was further confirmed that the weights of the identified 10 FCs in the LOOCV were significantly nonzero (two-sided Wilcoxon signed rank test, *p* < 0.001) demonstrating their important contribution to the classification of melancholic MDD and HC. In order to check for potential scanner or site-related effects, each subject’s weighted linear sum (WLS) was colored by site in the histogram of Fig. 2c.

**Table 2:** Identified 10 FCs for the melancholic MDD classifier.

ID	Name	Lat.	BSA atlas (Sulcus)	BA	r MDD	r HC	Weight	Contribution
1	Inferior Frontal Gyrus opercular part	L	Diagonal ramus of the lateral fissure	44	−0.018	0.173	−5.17	0.987
	Dorsomedial Prefrontal Cortex, Supplementary Motor Area: SMA, Pre-SMA, Frontal Eye Fields	R	Median frontal sulcus	6, 8, 9				
2	Dorsolateral Prefrontal Cortex, Middle Frontal Gyrus	L	Intermediate frontal sulcus	46	0.123	−0.065	5.20	0.978
	Posterior Cingulate Cortex, Precuneus	L	Internal parietal sulcus	7, 23, 31				
3	Thalamus	L	Thalamus	—	0.050	0.208	−3.27	0.517
	Anterior Cingulate Cortex, Posterior Cingulate Cortex	R	Subcallosal sulcus	23, 24, 33				
4	Inferior Frontal Gyrus Triangular part	L	Inferior frontal sulcus	45	0.286	0.407	−3.75	0.454
	Inferior Frontal Gyrus opercular part	R	Inferior precentral sulcus	44, 6				
5	Lingual Gyrus	L	Anterior intralingual sulcus	18	0.166	0.072	4.08	0.384
	Middle Occipital Gyrus	R	Lobe occipital	19				
6	Caudate	L	Caudate	—	−0.167	−0.067	−2.97	0.297
	Cuneus	L	Cuneal sulcus	18				
7	Lingual Gyrus	L	Posterior intra-lingual sulcus	18	0.251	0.078	1.52	0.263
	ParaHippocampus, Fusiform Gyrus	R	Collateral fissure	30,37				
8	Middle Cingulate Cortex	L	Calloso-marginal posterior fissure	23	−0.073	−0.168	2.68	0.255
	Calcarine	L	Occipito-polar sulcus	17				
9	Inferior Temporal Gyrus	L	Median occipito-temporal lateral sulcus	20	−0.093	0.015	−1.41	0.152
	Post Central Gyrus	L	Central sulcus	3				
10	Inferior Temporal Gyrus	L	Median occipito-temporal lateral sulcus	20	0.053	0.135	−1.03	0.084
	Superior Parietal Gyrus	R	Superior parietal sulcus	7				

The averaged functional correlation value of MDDs (rMDD) and HCs (rHC) are shown for each FC. As the patient group was always the positive class in the classifier, the sign of weight was positive when rMDD > rHC, and negative when rMDD < rHC. Contribution was calculated as (rMDD - rHC) × weight.

### Top two functional connections with the highest contributions

**Figure 3:**
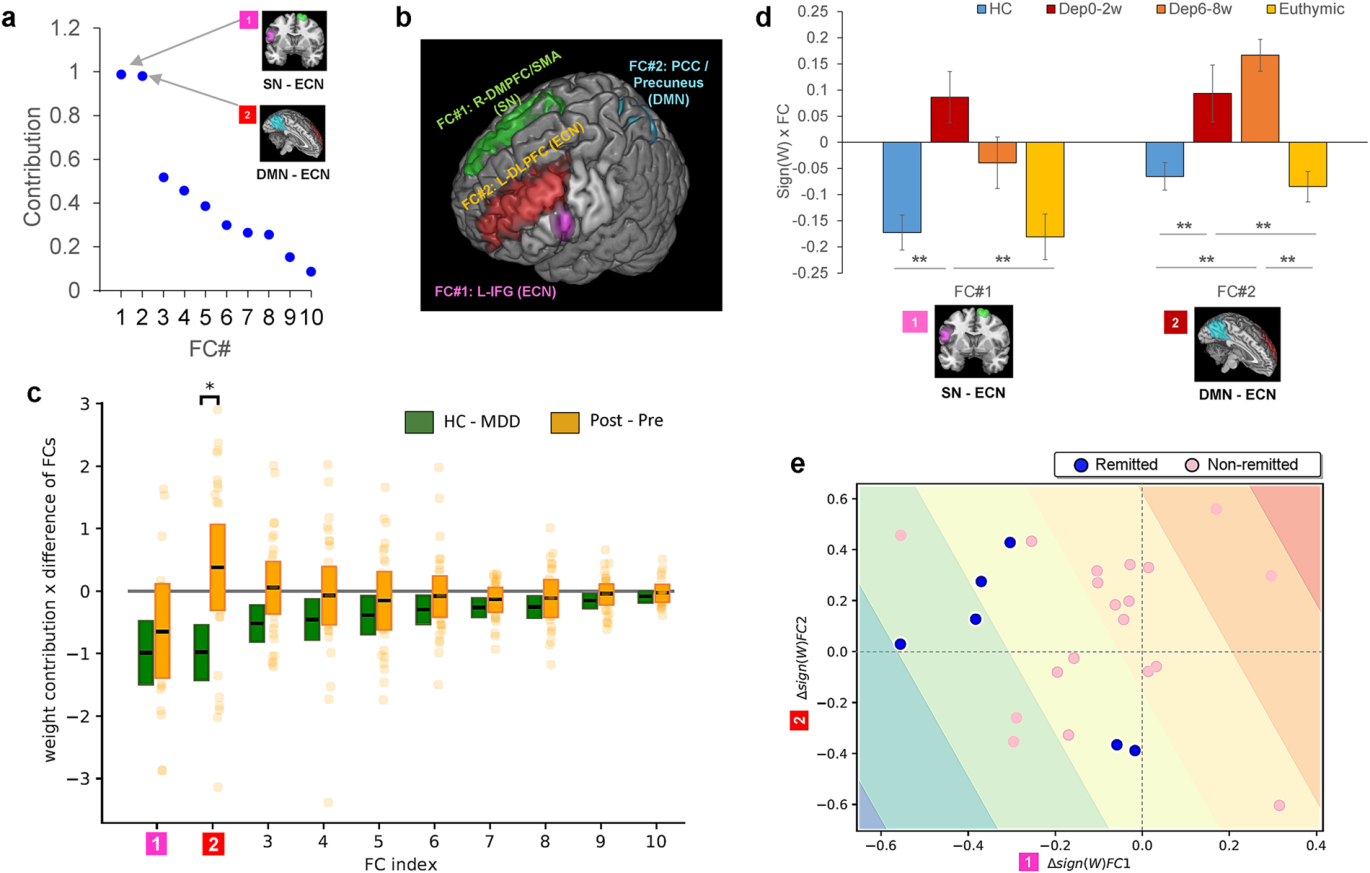
Individual functional connection changes from before to after antidepressant treatments. (**a**) Contribution of each of 10 FCs to the melancholic depression classifier. (**b**) Top two FCs with dominant contributions. There was a partially-overlapped region of both FCs in the left DLPFC. (The rendering tool: MRIcroGL 64-bit 12 June 2015, https://www.nitrc.org/projects/mricrogl) (**c**) For each FC, the post minus pre antidepressant treatment difference in average FC values of melancholic MDD (Post and Pre, n = 25) was compared with that difference between healthy control (HC, n = 65) and melancholic MDD (MDD, n = 65), and only the FC#2 showed significant difference (p < *0*.05). The box plot represents the 95% confidence interval around the mean. (**d**) FC#1 and FC#2 changes with the antidepressant treatment were examined. The bar graphs represent mean values with standard error (SE) bars. Compared to healthy control (HC, n = 65), pre-treatment (Dep0–2w, n = 25), post-treatment (Dep6–8w, n = 25), and an independent cohort of remitted depression patients (Euthymic, n = 34). (**e**) Changes in FC#1 and FC#2 for the 25 melancholic MDD patients with antidepressant treatments (blue dots: remit, n = 6, and pink dots: non-remit, n = 19). The blue to red gradient represents the probabilistic decision boundary of the logistic regression, where stronger blue (remit) and red (non-remit) colors are associated with a large probability of being in class and weaker colors represent increased uncertainty. *p < *0*.05, **p < 0.01.

We found outstanding contributions in the top 2 functional connections. Figure 3a shows the plot of each FC’s contribution to the classifier (i.e. multiplication of the classifier weight with the difference of each FC between patient and control groups). These top two FCs share an adjacent and part-overlapping brain region as one end of connection around the left DLPFC/IFG as shown in Fig. 3b. For the sign of these FCs, FC#1 showed less or no positive functional correlations in MDDs, compared to HCs who showed positive correlations between the ROIs of left inferior frontal gyrus (IFG opercular, BA44) in executive control network and right DMPFC (BA9)/frontal eye field (FEF, BA8)/supplementary motor area (SMA, BA6) in salience network. On the other hand, FC#2 shows positive functional correlations in melancholic MDDs, compared to HCs who have no or negative correlations between the ROIs of left dorsolateral prefrontal cortex (DLPFC, BA46) in executive control network and left posterior cingulate cortex (PCC)/Precuneus in default mode network.

### Effect of treatment with antidepressants

A subset of our melancholic MDD cohort (n = 25), who were treated with antidepressants as part of their routine clinical care (i.e., escitalopram in the majority of cases), had a further post-treatment rsfMRI scan at 6–8 weeks to evaluate the effect on functional connectivity. First, across these subjects, both the Beck Depression Inventory-II (BDI) score and the Hamilton Rating Scale for Depression (HAMD) were significantly reduced (Mean(SD): BDI Pre = 31.3 (7.48), BDI Post = 18.7 (12.2), paired t-test results on BDI: *p* = *7.26* × *10*^*-7*^; and HAMD Pre = 19.1 (5.63), HAMD Post = 11.3 (5.41), paired t-test results on HAMD: *p* = *7.29* × *10*^*-7*^).

Next, we evaluated the specific effect of antidepressant treatment on each FC of the melancholic MDD classifier. Based on the hypothesis that antidepressants would normalize the post-treatment MDDs’ FC values closer to those of HCs’, the difference in average FC values between post and pre antidepressant treatment was statistically compared to the difference between healthy control and MDD for each FC (Fig. 3c). FC values were multiplied by the sign of each weight to keep consistent directions; positive for MDD, negative for HC, and p-values were corrected using FDR. For each connection, p-value using Welch’s t test with FDR correction was as follows. FC#1: *p* = *0.328*, FC#2: *p* = *0.012*, FC#3: *p* = *0.083*, FC#4: *p* = *0.328*, FC#5: *p* = *0.328*, FC#6: *p* = *0.328*, FC#7: *p* = *0.328*, FC#8: *p* = *0.328*, FC#9: *p* = *0.328*, FC#10: *p* = *0.328*. The over-representation of the same values is because of the Benjamini-Hochberg FDR correction, and uncorrected p-value for each FC was as follows. FC#1: *p* = *0.318*, FC#2: *p* = *0.001*, FC#3: *p* = *0.016*, FC#4: *p* = *0.166*, FC#5: *p* = *0.312*, FC#6: *p* = *0.244*, FC#7: *p* = *0.162*, FC#8: *p* = *0.291*, FC#9: *p* = *0.181* FC#10: *p* = *0.327*. We found that all but two of the FCs were shifted in a normalized direction (i.e. in the direction of healthy controls) after the treatment, and the result of uncorrected p-value showed difference of treatment effects on two of the FCs. This could be because of heterogeneous influence of pharmacological treatment instead of regression toward the mean as a temporal change. However, one FC changed significantly in the opposite direction - away from that of healthy controls. Interestingly, this was the FC of left DLPFC and left PCC/Precuneus FC, which had the second highest contribution to the classifier (FC#2, pairwise comparison between the classifier contribution and the change by antidepressant treatments (*p* = *0.012*)). Note that in all panels of Fig. 3c–e, a positive change is in the direction of MDD, and a negative change is towards healthy control: this is because we multiply FC or differences of FC with the sign of the classifier weight in the melancholic MDD classifier.

Moreover, results of FC#1 (i.e., SN and ECN connectivity: SN-ECN) and FC#2 (i.e., DMN and ECN connectivity: DMN-ECN) changes with the antidepressant treatment were examined more in detail. In order to show early response and treatment outcome, all four conditions were plotted in Fig. 3d (healthy control (HC): n = 65, 0–2 weeks and 6–8 weeks treatment MDD (Dep0–2w, Dep6–8w for each period): n = 25, and an independent cohort of euthymic MDDs, who were fully remitted following long-term treatment with antidepressants (Euthymic: n = 34, Supplementary Table S1c). One way ANOVA yielded different significant variation among conditions for each FC (FC#1: *F(3,145)* = *7.37, p* = *0.000*, FC#2: *F(3,145)* = *1*0*.9, p* = *0.000)*. For FC#1, antidepressant treatments reasonably normalized the FC1 towards HC. Tukey’s HSD showed significant differences between HC and Dep0–2w (*p* = *0.008*), and Dep0–2w and Euthymic (*p* = *0.001*). The other contrasts were not significant: HC and Dep6–8w (*p* = *0.137, n.s*.), HC and Euthymic (*p* = *0.999, n.s*.), 0–2w and 6–8w (*p* = *0.331, n.s*.), and Dep6–8w and Euthymic (*p* = *0.173, n.s*.). On the other hand, for FC#2, there was no normalization effect observed in Dep6–8w. The same post-hoc test (Tukey’s HSD) kept showing significant differences not only between HC and Dep0–2w (*p* = *0.008*), but also between HC and Dep6–8w (*p* = *0.000*). In addition, there were significant differences between euthymic group and Dep0–2w (*p* = 0.008), and Dep6–8w (*p* = *0.000*) in the same way. The rest of conditions showed no significance: HC and Euthymic (*p* = *0.971, n.s*.), 0–2w and 6–8w (*p* = *0.606, n.s*.).

One might assume that there might be some compensatory relationship between FC#1 and FC#2. That is, more positive value in FC#2 might possibly help to have more negative value in FC#1. The answer was shown in Fig. 3e. No compensatory relationship was observed between FC#1 and FC#2 changes (*r* = *−0.147, p* = *0.483, n.s*.) Assuming that both FC#1 and FC#2 are equally contributing to prediction of remission, the blue to red gradient represents the probabilistic decision boundary of the logistic regression, where strong red and blue colors are associated with a large probability of being in the class of ‘non-remitted’ and that of’remitted’ respectively. Weaker colors represent increased uncertainty. Blue and pink dots show actually remitted (n = 6), and non-remitted (n = 19) melancholic MDD patients, and remission was assessed by HAMD score^38^.

### Application to non-melancholic MDD patients

**Figure 4:**
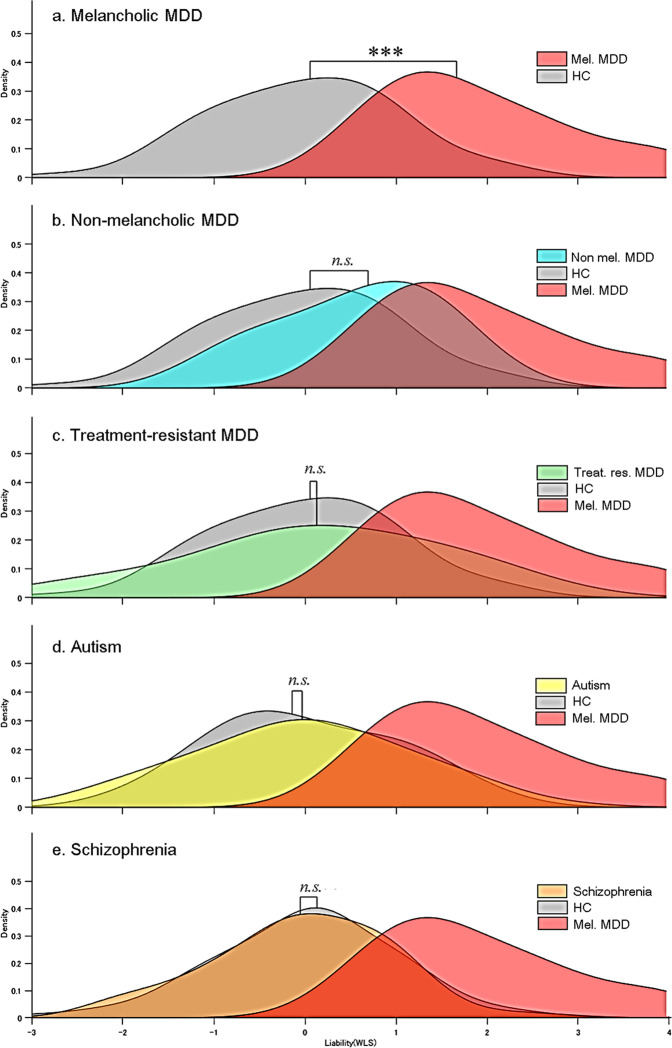
Generalization performance of the melancholic MDD biomarker to other subtypes and psychiatric disorders. To assess the difference in density distributions of WLS, Benjamini–Hochberg-corrected Kolmogorov–Smirnov test was applied to the comparison between each disorder and its healthy control. (**a**) Melancholic MDD (Mel. MDD) and healthy control (HC) were significantly different in density distribution of WLS (*p* = *6.3* ×   *10–14*). For other subtypes of MDD and psychiatric disorders, there was no significant difference was observed when the melancholic MDD classifier was applied; (**b**) non-melancholic MDD (Non Mel. MDD) and HC (*p* = *0.14*), (**c**) treatment-resistant MDD (Treat. Res. MDD) and HC (*p* = *0.81*), (**d**) autism and HC (*p* = *0.97*), and (**e**) schizophrenia and HC (*p* = *0.08*). ***p < *0.001*.

In order to examine a degree of diagnostic specificity of the melancholic MDD classifier, we also tested the classifier on 27 non-melancholic MDD and 27 additional control subjects. When it was applied to the non-melancholic MDD group, generalization accuracy was 69% (sensitivity 68%, specificity 71%, and AUC 0.72), suggesting that the classifier might be to some extent specific to melancholic MDD. As shown in the density distributions of the weighted linear sum (WLS), the distribution of melancholic MDD and healthy control were significantly different (Fig. 4a*p* = *6.3* × *10–14*, Benjamini–Hochberg-corrected Kolmogorov–Smirnov test), and non-melancholic MDD appeared to be shifted toward the direction of the healthy control group along the axis of the melancholic MDD classifier, but this did not reach significance level (Fig. 4b, *p* = *0*.*14*, Benjamini–Hochberg-corrected Kolmogorov–Smirnov test).

### Application to treatment-resistant MDD

In addition to the non-melancholic MDD patients, we applied the classifier to an independent cohort of treatment-resistant MDD (16 patients, 16 controls). The accuracy was 72% (sensitivity 69%, specificity 75%, and AUC 0.68) and there was no significant difference between the patient and control distributions (Fig. 4c, *p* = *0.81*, *n.s*., Benjamini–Hochberg-corrected Kolmogorov–Smirnov test).

### Application to other psychiatric disorders

To further evaluate the specificity of the classifier, we utilized two other psychiatric cohorts: autism spectrum disorder (ASD; 74 patients, 36 controls) and Schizophrenia spectrum disorder (68 patients, 102 controls). For the ASD cohort, accuracy was 55% (sensitivity 53%, specificity 61%, and AUC 0.63), and as illustrated in the density distributions, there was no difference between patients and controls (Fig. 4d, *p* = *0.97*, *n.s*., Benjamini–Hochberg-corrected Kolmogorov–Smirnov test). In the schizophrenia spectrum disorder cohort, accuracy was 41% (sensitivity 40%, specificity 41%, and AUC 0.44). In fact, the schizophrenia spectrum disorder patients’ distribution was located significantly on the opposite side to MDD with respect to healthy control (Fig. 4e, *p* = *0.08*, *n.s*., Benjamini–Hochberg-corrected Kolmogorov–Smirnov test). Overall, these results support a degree of diagnostic specificity of the melancholic MDD classifier. Generalization of the ASD classifier and the Schizophrenia classifier to our MDD cohort was examined and reported elsewhere^37,39^.

## Discussion

The purpose of this study was to develop a melancholic MDD biomarker, and to explore its sensitivity to treatment. In particular, we aimed to test whether functional connectivity changes were influenced homogenously, or heterogeneously, as a window into understanding why treatments might have a limited effect, and potentially even identifying candidate targets for future treatment. Ten FCs were selected by our sparse machine learning algorithm as the melancholic depression biomarker. The validity of the melancholic MDD biomarker was demonstrated by generalization to a completely independent validation cohort, and its specificity was demonstrated by the following applications of the classifier to other subtypes and psychiatric disorders. Moreover, this study showed that antidepressants had a heterogeneous effect on the functional connectivity underlying depression, and this biomarker allowed us to identify and localize the effect of antidepressant action on different functional connections. Specifically, it was highlighted that the FC with the second highest contribution did not show significant normalization after 6–8 weeks of antidepressant treatments, whereas the rest of the FCs were normalized.

Out of ten FCs, the top two FCs with outstanding contributions in the melancholic depression classifier included the FCs with left IFG in ECN and right DMPFC/FEF/SMA in SN for FC#1, and left DLPFC/IFG in ECN and PCC/Precuneus in DMN for FC#2. Although it is still difficult to define their precise neuropsychological roles in depression, previous studies provide some clues. These regions have been associated with cognitive flexibility, for instance, assessed with reversal learning tasks^40,41^, in which depression patients typically have functional deficits in. Regarding FC#1 (SN-ECN), previous study showed that increased neural activity in IFG and DMPFC were associated with empathic accuracy with compassion meditation training^42^, and reduced connectivity between DMPFC-IFG was observed in depressed adolescents during cognitive reappraisal of emotional images^43^. In addition, priming-TMS studies shows that the DMPFC has a causal role in forming social-relevant impression including face-adjective pair, and processing verbal emotional stimuli^44,45^. As for FC#2 (DMN-ECN), bilateral DLPFC/IFG have been associated with attention control and conflict processing^46^, and PCC/Precuneus have been associated with anhedonic depression and anxious arousal^47^.

From a general perspective, the correlation between a drug and a functional connection could arise for any one of three reasons: as a cause, epiphenomenon, or compensation for other changes observed in the disorder. In the case of the left DLPFC/IFG - PCC/Precuneus, the second and third possibilities seem unlikely for the following reasons. The epiphenomena hypothesis assumes that the increase or decrease of FC#2 (DMN-ECN) could occur simultaneously with depressive symptoms but not directly in causal relationships. As a remedy for depression, antidepressant therapy reduces severity of depression (i.e., BDI, HAMD scores). Although this hypothesis predicts decrease in FC#2 (DMN-ECN), this is against our experimental observation (Fig. 3d). The compensation hypothesis assumes that the FC#2 (DMN-ECN) increase actively compensates deteriorating effects induced by the abnormal changes in other FCs in depression, including FC#1 (SN-ECN) increase. This hypothesis predicts positive correlations between changes in FC#1 (SN-ECN) and FC#2 (DMN-ECN), but we did not observe the predicted positive correlations after antidepressants therapy (Fig. 3e).

On one of the recently suggested reasons on why antidepressants including SSRI might not seem to affect the FC#2 (DMN-ECN) immediately, there was a review paper pointing a problem of antidepressant treatment using SSRI^48^. The authors suggested that the depression symptom reduction is not achieved by the direct pharmacological properties of SSRI, but by the brain’s compensatory responses to restore homeostasis. That may explain why it takes several weeks to achieve the SSRI treatment response outcome rather than 30 minutes after taking the medicine like aspirin. This hypothesis cannot be directly applied to our fMRI results, because the previous PET study showed human serotonin transporter exists more in midbrain^49^ rather than in cerebral cortex. However, it could be indirectly associated with our results, showing that the FC#2 (DMN-ECN) moved to the opposite direction of normalization once, and then gradually coming back to the normalizing direction after entering into the euthymic state. It is impossible to deny the possibility of placebo effect in this paper, because we do did not have any appropriate control group as this was an observational study, not a clinical trial. The potential confounding component here is the time course effect which would make the FCs to regress toward the mean. Although this is the largest limitation, for example, if our results could just be a placebo effect or due to uniform temporal changes of functional correlations, then, all the functional connections should most probably regress toward the same direction to normalization, as BDI and HAMD depression symptom scores were significantly improved on average. However, our data showed different functional connection changes among the identified 10 FCs as shown in Fig. 3c (eight FCs to normalization, one FC with no change, and one FC to the opposite direction). Therefore, though it is the largest limitation that there is no placebo group in this study, there would be a high possibility that our data were able to examine the functional connection specific pharmacological effects.

In addition to a wealth of correlative evidence that links left dorsolateral prefrontal cortex connections to depression^24,50–57^, there is other evidence that suggests an active functional relationship. First, it is an established target for repetitive transcranial magnetic stimulation (rTMS)^58–60^ therapy for depression. Second, recent neurofeedback treatment directly targeting FC#2 (DMN-ECN) connectivity has been found to be effective^61–63^. Therefore although more studies are needed, the current evidence points toward a causal influence of DLPFC connectivity and depression.

## Limitations

There were several limitations in this study. Given the highly heterogeneous nature, it is important to note limitations of the interpreting rsfMRI results in depression. First, as a limitation of resting state data, we cannot control some spontaneous thoughts or mental activity, and this also could affect stability of the data. Second, although we specifically focused on melancholic depression and identified 10 FCs, this may still limit high classification accuracy^64^ as some previous papers suggest the effectiveness of an additive model of categorical diagnosis and symptom dimensions^65^. In addition, we had a small sample size of independent drug-free cohort (with neither antidepressant nor antipsychotic use for more than one month). The most critical limitation in this study was that antidepressant effects were tested on relatively small size of MDD cohort with pre- and post- treatments, and we did not have a placebo or no-treatment group because of ethical issues and difficulty in recruitment. This needs to be tested on a larger cohort in the future. However, the top two critical FCs with especially high contributions included the brain regions which have been persistently implicated across previous depression studies. Ideally, future studies need to track the longer-term profile of FC#2 (DMN-ECN) in larger data sets. Although FC#2 (DMN-ECN) was still abnormal at 6–8 weeks after antidepressant treatments, it was normalized in the euthymic group, which might include only the patients who are responsive to treatments though.

## Conclusions

This study provides novel evidence on the importance of the critical functional connection between PCC/Precuneus and left DLPFC/IFG (DMN-ECN), which did not show any improvement right after the antidepressant treatments. Our data not only validated the development of rsfMRI-based melancholic depression biomarker in understanding mechanisms of disorder and treatment, but also suggested the possibility that combined therapy of antidepressants and targeted neurostimulation/neurofeedback could be an optimal strategy to pursue in the future therapeutic studies.

## Supplementary information

Supplementary information.

## Data Availability

We are the registered member of Decoded Neurofeedback (DecNef) Project Brain Data Repository (https://bicr-resource.atr.jp/decnefpro/), and the data for this study are available from the website on reasonable request by qualified researchers.
