# Publisher Correction: Primary functional brain connections associated with melancholic major depressive disorder and modulation by antidepressants

**DOI:** 10.1038/s41598-020-73436-y

**Published:** 2020-10-14

**Authors:** Naho Ichikawa, Giuseppe Lisi, Noriaki Yahata, Go Okada, Masahiro Takamura, Ryu-ichiro Hashimoto, Takashi Yamada, Makiko Yamada, Tetsuya Suhara, Sho Moriguchi, Masaru Mimura, Yujiro Yoshihara, Hidehiko Takahashi, Kiyoto Kasai, Nobumasa Kato, Shigeto Yamawaki, Ben Seymour, Mitsuo Kawato, Jun Morimoto, Yasumasa Okamoto

**Affiliations:** 1 Department of Psychiatry and Neurosciences, Graduate School of Biomedical Sciences, Hiroshima University, Hiroshima, Japan; 2 ATR Brain Information Communication Research Laboratory Group, Kyoto, Japan; 3 Institute for Quantum Life Science, National Institutes for Quantum and Radiological Science and Technology, Chiba, Japan; 4 Medical Institute of Developmental Disabilities Research, Showa University, Tokyo, Japan; 5 Department of Functional Brain Imaging Research, National Institutes for Quantum and Radiological Science and Technology, Chiba, Japan; 6 Department of Neuropsychiatry, Keio University School of Medicine, Tokyo, Japan; 7 Department of Psychiatry, Kyoto University Graduate School of Medicine, Kyoto, Japan; 8 Graduate School of Medical and Dental Sciences, Tokyo Medical and Dental University, Tokyo, Japan; 9 Department of Youth Mental Health, Graduate School of Medicine, The University of Tokyo, Tokyo, Japan; 10 Computational and Biological Learning Lab, Cambridge University, Cambridge, UK

Correction to: *Scientific Reports* 10.1038/s41598-020-60527-z , published online 26 February 2020.

The original version of this Article contained errors.

In the original PDF version, the published date was omitted and now reads:

“Published: 26 February 2020”.

In addition, there were typographical errors in the Methods section, under the subheading “Experimental protocol and data acquisition”, where:

“In the scan room with dimmed lights, participants were not required to keep looking at a cross mark in the center of the monitor screen, think of anything in particular, and not to sleep.”

now reads:

“In the scan room with dimmed lights, participants were required to keep looking at a cross mark in the center of the monitor screen, think of nothing in particular, and not to sleep.”

In Table 2 where the ‘Contribution’ value for ID #7 was incorrect, the value “\” now reads “0.263”.

In the Results section under the subheading “Effect of treatment with antidepressants”, where BDI and HAMD values were reversed,

“(Mean(SD): BDI Pre = 19.1 (5.63), BDI Post = 11.3 (5.41), paired t-test results on BDI: *p* = 7.29 × 10^−7^; and HAMD Pre = 31.3 (7.48), HAMD Post = 18.7 (12.2), paired t-test results on HAMD: *p* = 7.26 × 10^−7^).”

now reads:

“(Mean(SD): BDI Pre = 31.3 (7.48), BDI Post = 18.7 (12.2), paired t-test results on BDI: *p* = 7.26 × 10^−7^; and HAMD Pre = 19.1 (5.63), HAMD Post = 11.3 (5.41), paired t-test results on HAMD: *p* = 7.29 × 10^−7^).”

In the Supplementary Information file, there were missing values in Table S1c under the “Time 2 (6-8w)” column and on the “Severity of symptoms (BDI-II) “and “Observer-rated depression scale (HAMD-17)” rows. These values now read “18.7 (12.2)” and “11.3 (5.4)” respectively.

These errors have now been corrected in the PDF and HTML versions of the Article, and in the accompanying Supplementary Information file.

