# Supplementary material for: Primary functional brain connections associated with melancholic major depressive disorder and modulation by antidepressants
Source: Sci Rep. 2020 Feb 26;10:3542. doi: 10.1038/s41598-020-60527-z (PMC7044159; doi:10.1038/s41598-020-60527-z)

Supplementary information for “Primary functional brain connections associated with melancholic major depressive disorder and modulation by antidepressants”

Naho Ichikawa^1^, Giuseppe Lisi^2^, Noriaki Yahata^3^, Go Okada^1^, Masahiro Takamura^1^, Ryu-ichiro Hashimoto^4^, Takashi Yamada^2^, Makiko Yamada^3,5^, Tetsuya Suhara^3^, Sho Moriguchi^6^, Masaru Mimura^6^, Yujiro Yoshihara^7^, Hidehiko Takahashi^7,8^, Kiyoto Kasai^9^, Nobumasa Kato^4^, Shigeto Yamawaki^1^, Ben Seymour^2,10^*, Mitsuo Kawato^2^, Jun Morimoto^2^, & Yasumasa Okamoto^1^*

*oy@hiroshima-u.ac.jp

^1^Department of Psychiatry and Neurosciences, Graduate School of Biomedical Sciences, Hiroshima University, Hiroshima, JAPAN; ^2^ATR Brain Information Communication Research Laboratory Group, Kyoto, JAPAN; ^3^Institute for Quantum Life Science, National Institute of Radiological Sciences, National Institutes for Quantum and Radiological Science and Technology, Chiba, JAPAN; ^4^Medical Institute of Developmental Disabilities Research, Showa University, Tokyo, JAPAN; ^5^Department of Functional Brain Imaging Research, National Institutes for Quantum and Radiological Science and Technology, Chiba, JAPAN; ^6^Department of Neuropsychiatry, Keio University School of Medicine, Tokyo, JAPAN; ^7^Department of Psychiatry, Kyoto University Graduate School of Medicine, Kyoto, JAPAN; ^8^Graduate School of Medical and Dental Sciences Tokyo Medical and Dental University, Tokyo, JAPAN ^9^Department of Youth Mental Health, Graduate School of Medicine, The University of Tokyo, Tokyo, JAPAN; ^10^Computational and Biological Learning Lab, Cambridge University, Cambridge, UK.

*Supplementary Information*

**Supplementary Methods.**

Feature selection with reduction of nuisance variable effects

Application to non-melancholic MDD, treatment-resistant MDD.

Application to other psychiatric disorders.

Application to euthymic MDD.

Changes of each FC: post minus pre antidepressant treatments.

**Supplementary Results.**

Tables S1: Demographic information of the participants

Tables S2: Scanner information and resting-state fMRI protocols

Tables S3: Head motion during resting-state

Figure S1: Generalization to an independent cohort assessed by permutation tests

**Supplementary Methods.**

**Feature selection with reduction of nuisance variable effects**

As specific steps, in the first data matrix X1 of demographic information, the column of diagnosis contained either 1 or 0 (i.e., MDD or HC). The next four columns of scanner/site contained either [1 0 0 0] for the Site1, [0 1 0 0] for the Site2, [0 0 1 0] for the Site3, or [0 0 0 1] for the Site4. The sixth column was for age, and the seventh column was for sex (i.e., 1 for male and 0 for female). The second data matrix X2 pools the off-diagonal lower-triangular elements of correlation matrix as the FC of a single subject. By applying L1-SCCA to these data, the sparse projection matrices of V1 and V2 were derived from the pair of data matrices (i.e., X1 and X2). We here defined the diagnostic canonical variables and the diagnostic canonical constraint, and which should be associated only with the diagnostic label. We used the columns of V1 that have non-zero elements only in the row corresponding to the diagnostic label, and only the columns of V2 corresponding to the diagnostic canonical variables based on the sum of absolute value across columns as union of features across diagnostic canonical variables. This means, the columns which had non-zero values in the rows corresponding to the scanner/site, age, sex were disregarded. In this way, identifying the FCs corresponding to the diagnostic canonical variables enabled us to select only essential FCs for MDD/HC classification, and simultaneously reduced undesirable effects of nuisance variables (i.e., scanner/site, age, sex).

**Application to non-melancholic MDD, treatment-resistant MDD.**

In order to test if the classifier was specific to the characteristics of melancholic MDD, we applied the same classifier to the datasets of non-melancholic and treatment-resistant MDD (Demographics are shown in **Supplementary Table S1b**, with two MDD patients lacking MINI scores of previous episodes, melancholia, and comorbidity). Non-melancholic MDDs are from the all MDD dataset with more than mild depressive state (BDI score > = 17).

**Application to other psychiatric disorders.**

Autism spectrum disorder (ASD) dataset was adopted from our previous investigation. In order to minimize any effect from comorbidity of depression, the autism spectrum disorder dataset was limited to the data with no active antidepressant medication. A total of 110 participants included 74 ASD patients (No. of male/female = 58/16, Age (year): mean (SD) = 31.5 (8.5)) and 36 healthy controls (No. of male/female = 30/6, Age (year): mean (SD) = 30.9 (6.9)).

For the schizophrenia spectrum disorder (SSD) data (Yoshihara et al., in submission), all participants provided written informed consent that was approved by the Committee on Medical Ethics of Kyoto University. A total of 170 participants included 68 SSD patients (No. of male/female = 33/35, Age (year): mean (SD) = 38.4 (9.1), 64 Schizophrenia and 4 Schizoaffective disorder, Duration of illness (year): mean (SD) = 12.8 (7.8)) and 102 healthy controls (No. of male/female = 62/40, Age (year): mean (SD) = 31.1 (9.6)). See the **Supplementary Table S1** for details of MRI experimental settings for each site.

**Application to euthymic MDD.**

Thirty-six patients who are in euthymic states for more than two months were recruited, and their demographic information was shown in **Supplementary Table S1c**. Their resting state fMRI and T1 structural data were scanned using the identical scan protocols at the Site 4, and went through the identical preprocessing steps.

**Changes of each FC: post minus pre antidepressant treatments.**

For the analysis of pre-post treatment effects, we computed contribution scores of each FC using the following equations:


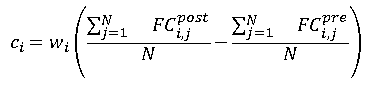


Equation (1)

where N is the number of patients who underwent the treatment and
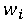
is the classifier weight. In order to have a point of reference, we computed the same contribution score, for the MDD and healthy control populations:


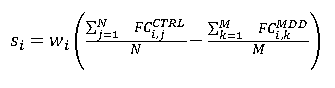


Equation (2)

where M is the total number of MDD patients, and N is the number of healthy control subjects. The
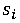
 represents the difference in average
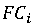
 between healthy controls and MDD, weighted by the classifier’s weight. It should be noted that the
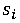
should always be negative since MDD is the positive class in the classifier, and
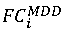
 is the subtrahend (i.e. negative sign in the subtraction) in Formula 2. The
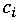
 represents the difference in average
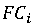
 between post- and pre- antidepressant treatments. Negative value of
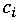
 (i.e. post- minus pre- treatment) means that the post-treatment
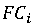
 is closer to healthy controls, while positive value means that post-treatment
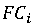
 is closer to MDD. For each
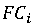
, we compared
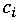
 and
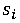
 using the Welch’s t-test, due to the different sample sizes between HCs, MDDs, and the MDD patients who underwent treatment:


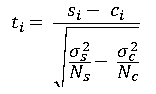


Equation (3)

where
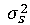
 is the sample variance of the
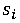
 , and it is obtained using the *variance sum law* as the sum of the variances of
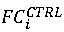
$\mathrm{FC}_{i}^{\mathrm{CTRL}}$and
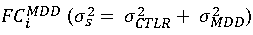
. Similarly, $\sigma_{c}^{2}$
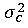
is computed as the sum of variances of
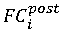
$\mathrm{FC}_{i}^{\mathrm{post}}$and
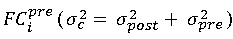
. The sample sizes are computes as
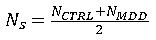
$N_{s}=\frac{N_{\mathrm{CTRL}}+N_{\mathrm{MDD}}}{2}$and
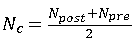
$N_{c}=\frac{N_{\mathrm{post}}+N_{\mathrm{pre}}}{2}$. The P value of each test was corrected for multiple comparisons by the Benjamini–Hochberg procedure.

**Supplementary Results.**

**Table S1:** Demographic information of the melancholic MDD classifier.


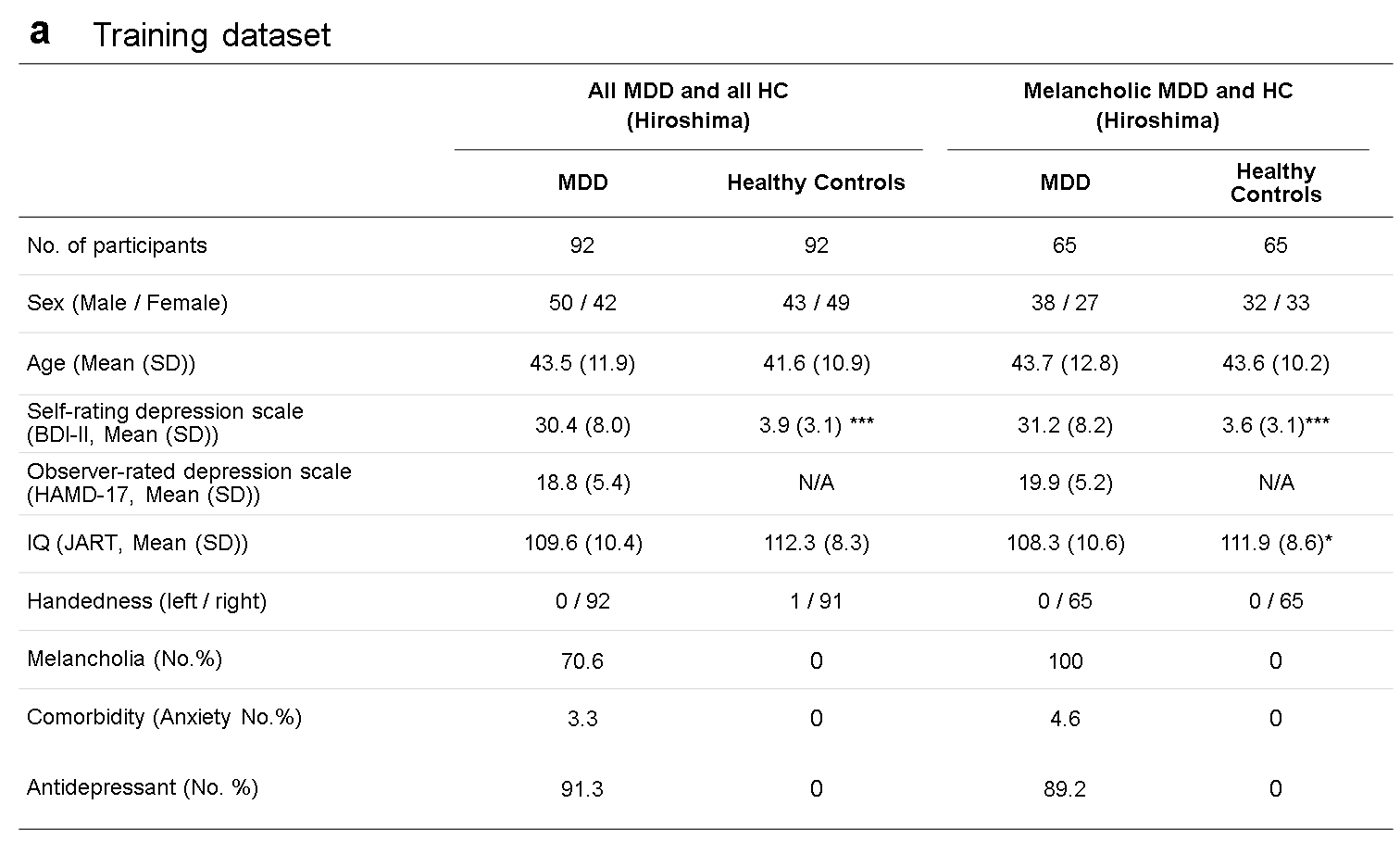


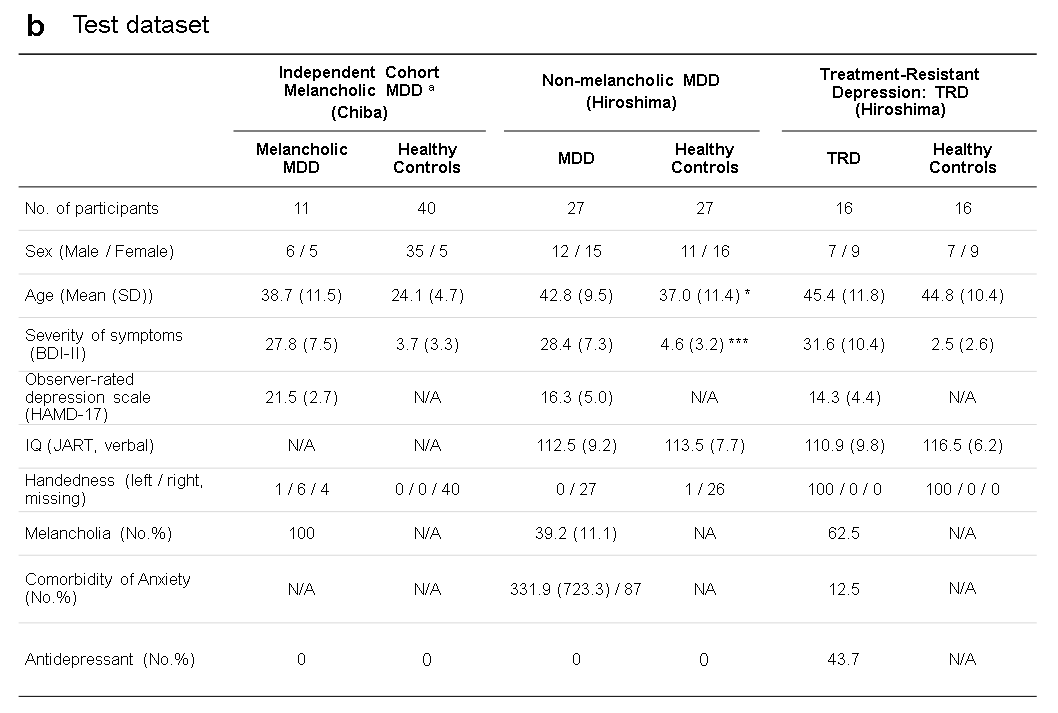


**
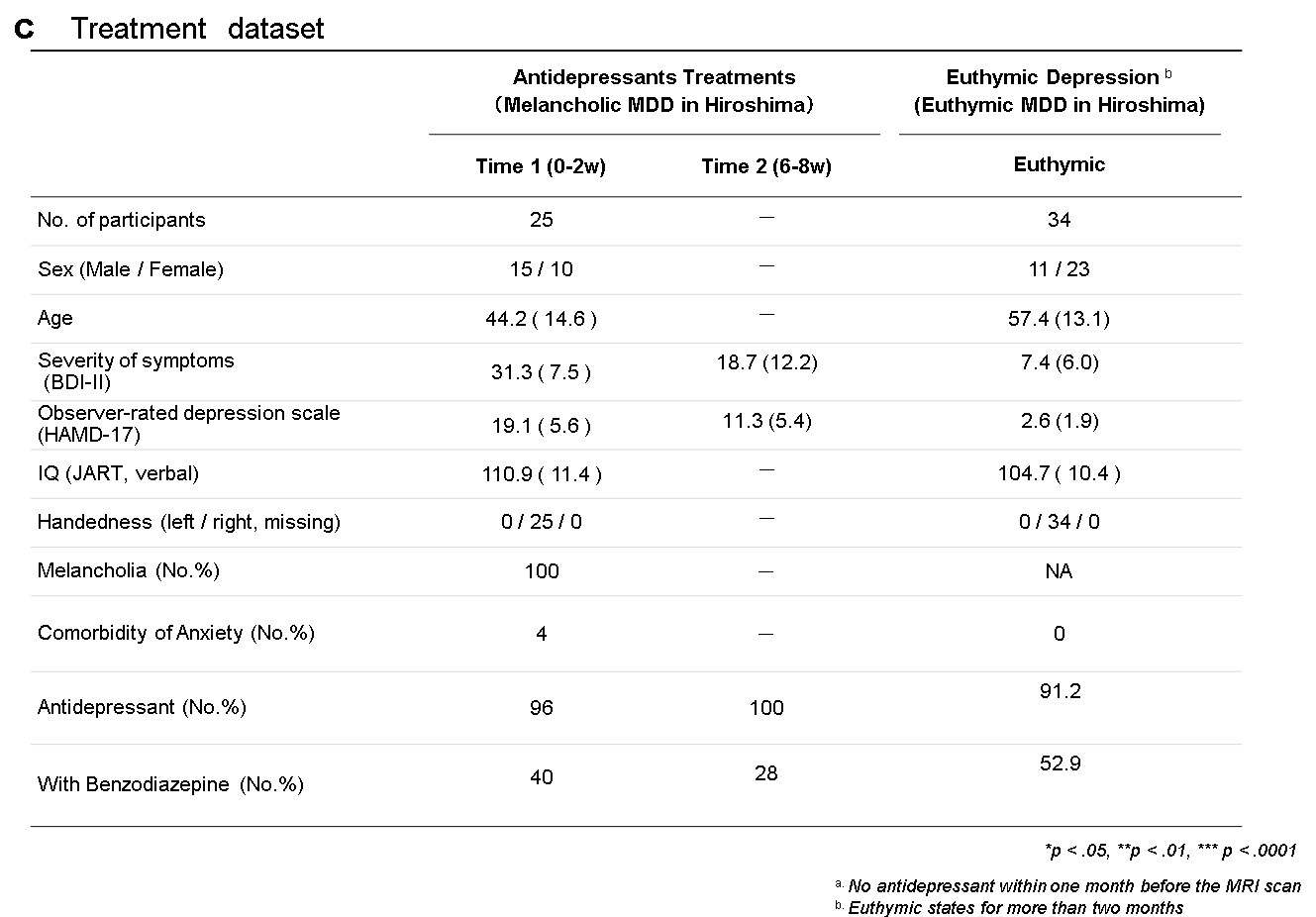
**

**Table S2:** Scanner information and resting-state fMRI protocols of melancholic MDD, healthy control, other subtypes of MDD, autism spectrum disorder, and schizophrenia. All the euthymic MDD data shown in Table S1c was collected at the Site 4 with identical settings.


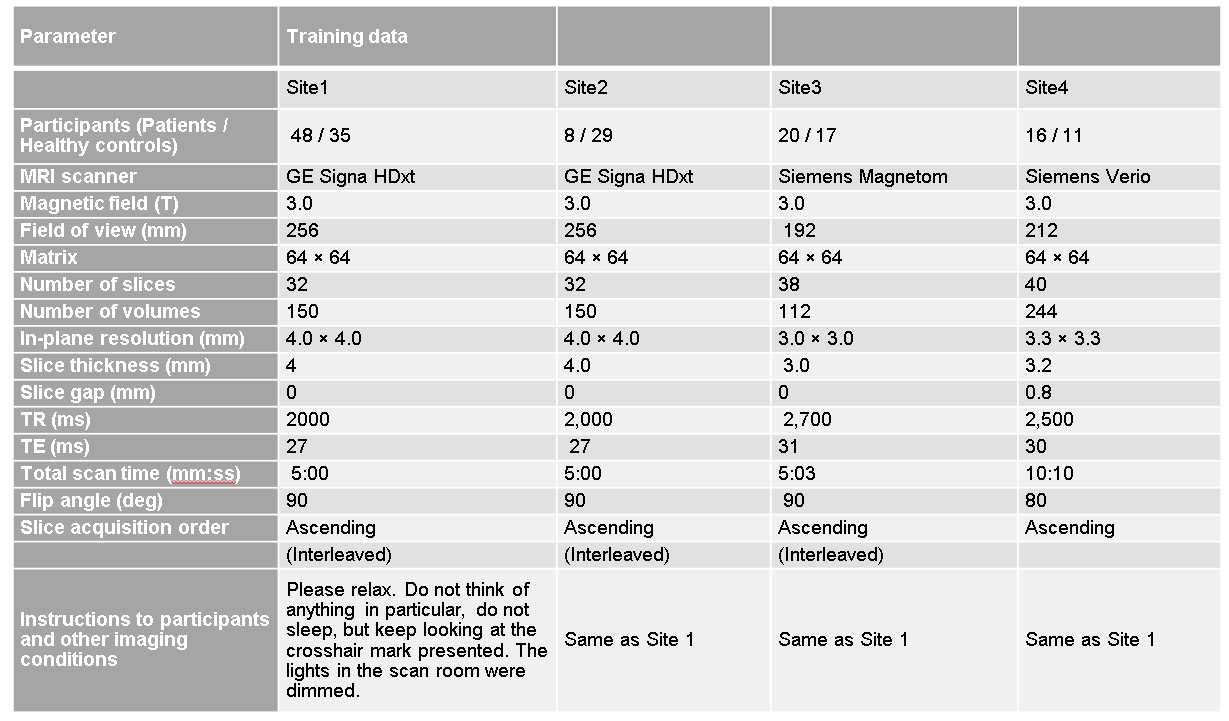


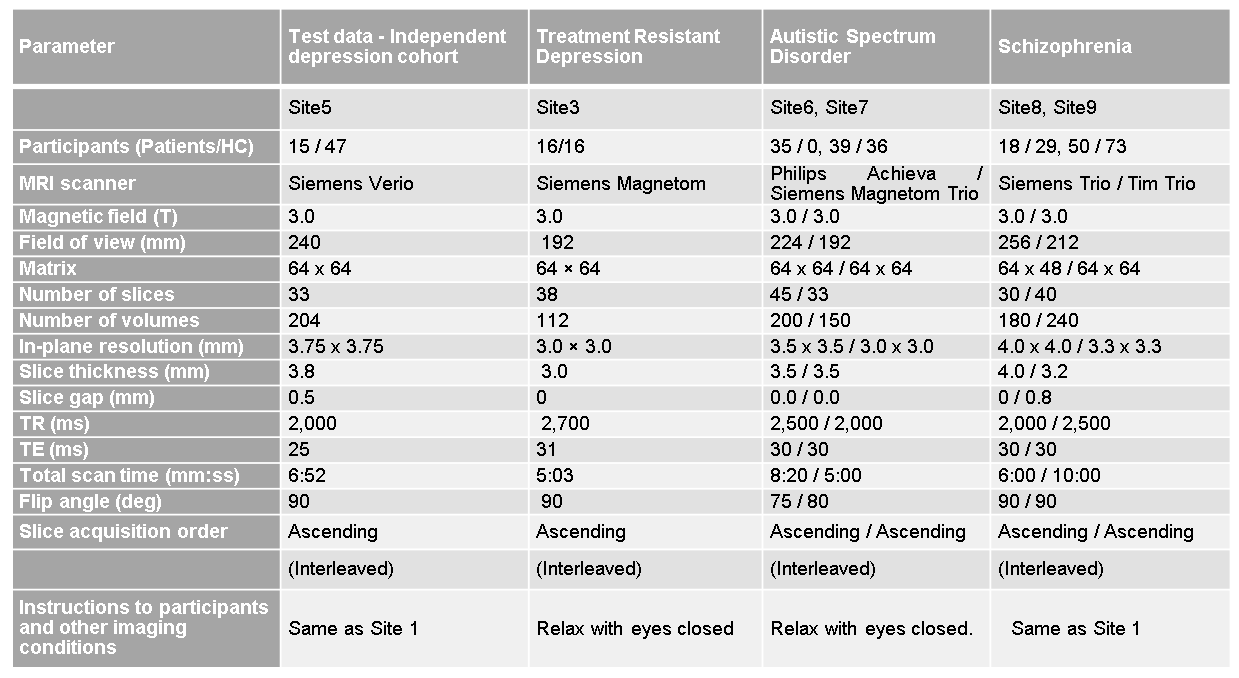


**Table S3:** Head motion of MDD and healthy control in the training dataset and test dataset.


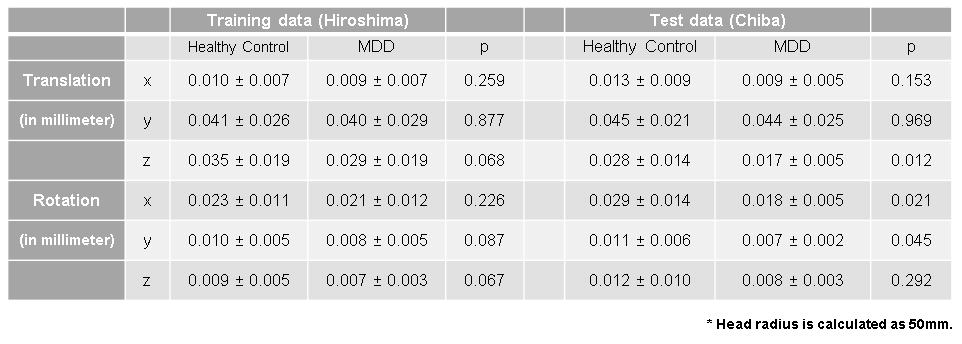


**Figure S1.** Classification results of the melancholic MDD biomarker and its generalization performance to other subtypes and psychiatric disorders. Density of the weighted linear sum computed based on the melancholic MDD biomarker for a.) training dataset (melancholic MDD n = 65, healthy control: n = 65) and b.) test dataset from an independent site (melancholic MDD: n = 11, healthy control: n = 40). Permutation tests show the histogram of the permutation test (1,000 repetitions) for c) the training dataset LOOCV, and show d.) a completely independent test dataset accuracies, and the binomial distribution is shown as a green curve. The accuracies of the melancholic MDD classifier trained and tested without permutation were shown as red vertical lines. The results of permutation test were significant for LOOCV (*p = .002*) and for the independent test dataset (*p = .040*). ** p < .05, *** p < .005.*


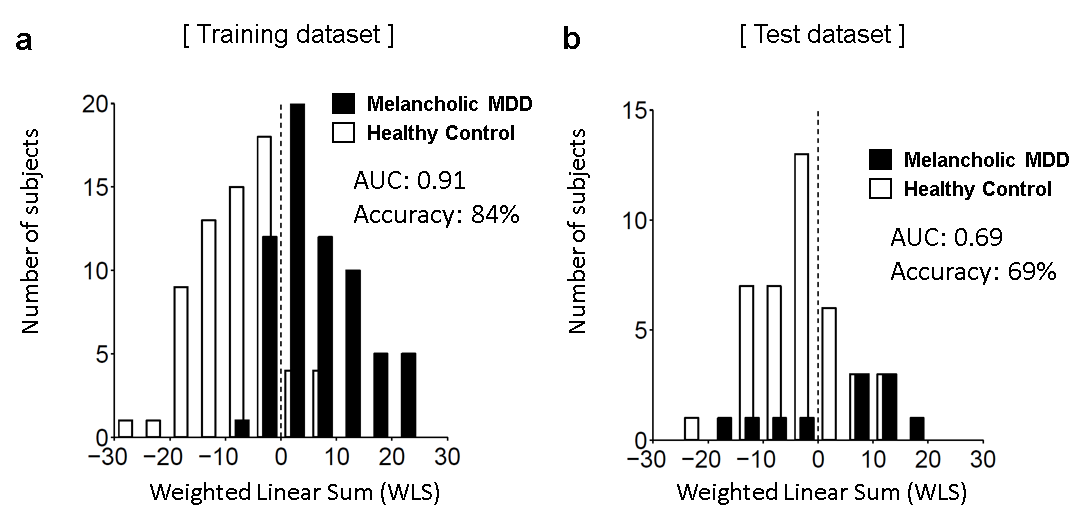


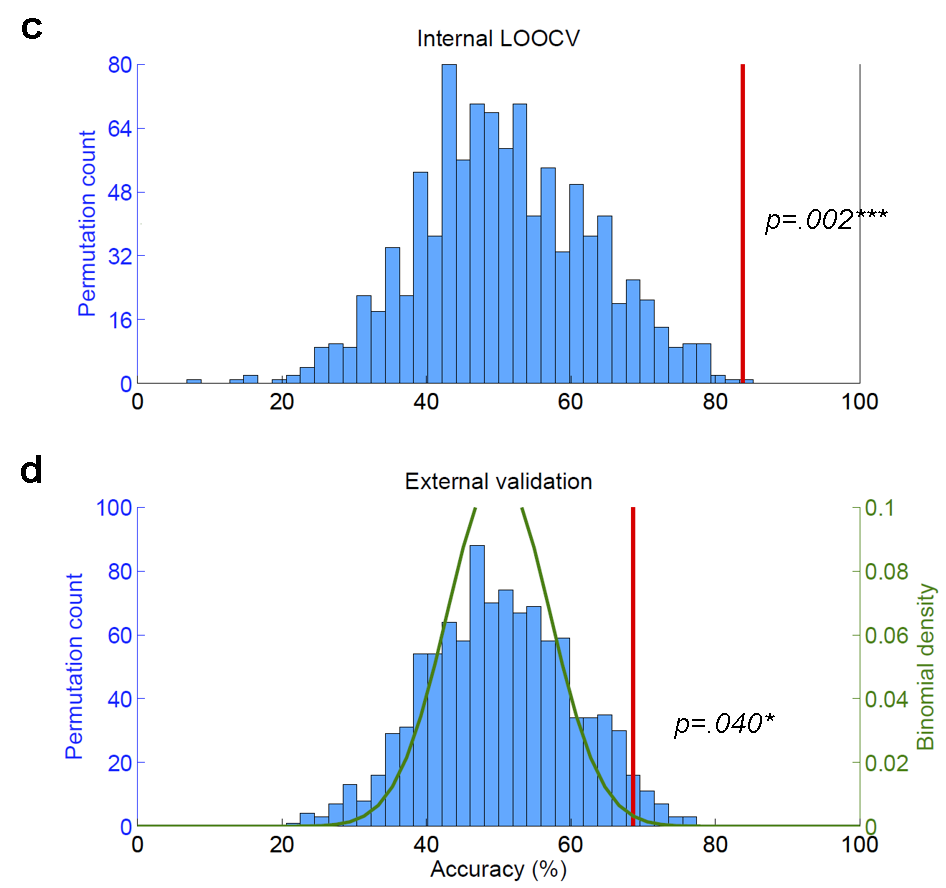

Supplement: Supplementary file 1 — Supplementary information. [file 41598_2020_60527_MOESM1_ESM.docx]
